# Causes of death in patients infected with HIV from 1985 to 2008

**DOI:** 10.1186/1758-2652-13-S4-P222

**Published:** 2010-11-08

**Authors:** J Mendez, R Sarmento e Castro, AR Domingues da Silva

**Affiliations:** 1 Hospital de Joaquim Urbano, Rua Câmara Pestana, 348, Porto, Portugal

## Introduction

Death rates and causes of death in patients infected with HIV seem to be related to the kind of ARV therapy they have been treated with and have altered the causes of death along the years.

## Purpose of the study

To study the causes of death in HIV patients in the era before HAART, partial use of HAART, and HAART.

## Methods

Retrospective study of the clinical history of the HIV patients who died in the HJU since 1985. The patients were dived into three periods in accordance with the kind of therapy: 1985 to 1995 (pre- HAART), 1996 to 1999 (partial use of HAART), and 2000 to 2008 (HAART). To accurately evaluate the causes of death 160 patients were eliminated as their causes of death have not been clearly conclusive.

## Summary of results

Between October 1985 and December 2008, a total of 4362 HIV infected patients were examined in the HJU, of which 936 (21.05%) died. Of these 936, 85.3% were males, and 14.7% were females. The average age at the time of death was 36.8 (youngest 17, and oldest 80 years old). The average duration of the infection was 41.76 months. The identified mean of transmission were intravenous drug use (67.3%), heterosexuality (17.2%), homosexuality (6.5%), blood derivatives transfusions (0.3%), and in 8.7% of the patients the risk behaviour was unknown. Among all the dead patients, 32.5% were exclusively HIV infected, 35.7% HIV/HCV, 22.6% HIV/HCV/HBV, and 9.2% HIV/HBV. The average CD4 nadir value was 91/mm^3^, and at the date of death was 112.2/mm3. The average viral load at the date of death was 147.837 cop/ml. During the period 1985/1999 the death rate was 20.04%. During the period 1996/2000 this rate was 11.21%, and in the last 8 years it was 13.76%. As to the causes of death, 776 clinical histories have been studied.

## Conclusions

We noticed a significant decrease of death rates between the first and the last years of HIV infection which is a consequence of the use of a more efficient therapeutics. The introduction of the use of HAART resulted in a relative decrease of deaths due to opportunistic infections and in a relative increase of deaths due to severe hepatic diseases, non opportunistic infections and malignancies.

